# The Value of Early Positive Nucleic Acid Test and Negative Conversion Time of SARS-CoV-2 RNA in the Clinical Outcome of COVID-19 Patients

**DOI:** 10.3389/fmed.2022.826900

**Published:** 2022-04-28

**Authors:** Xin Zang, Liangkun Xiong, Junyao Zhu, Fangfang Zhao, Shihong Wang, Wenhui Zeng, Kaihuan Yu, Yongzhen Zhai

**Affiliations:** ^1^Department of Infectious Disease, Shengjing Hospital of China Medical University, Shenyang, China; ^2^Department of Hepatobiliary Surgery, Renmin Hospital of Wuhan University, Wuhan, China; ^3^Department of Infectious Disease, Fujian Medical University Affiliated First Quanzhou Hospital, Quanzhou, China; ^4^Department of Paediatrics, Renmin Hospital of Wuhan University, Wuhan, China

**Keywords:** COVID-19, SARS-CoV-2, nucleic acid test, negative conversion, clinical characteristics

## Abstract

**Background:**

The outbreak of coronavirus disease (COVID-19) poses a great threat to global public health. At present, the number of newly confirmed COVID-19 cases and deaths is increasing worldwide. The strategy of comprehensive and scientific detection of severe acute respiratory syndrome coronavirus 2 (SARS-CoV-2) through quantitative real-time polymerase chain reaction (qRT-PCR) for special populations and environments provides great support for the prevention and control of this pandemic in China. Our study focused on determining the factors associated with the length of time from symptom onset to the first positive nucleic acid test of throat swabs in COVID-19 patients, evaluating the effect of early positive nucleic acid detection on the disease severity and its significance in prognosis, and predicting the factors associated with the time from positive SARS-CoV-2 RNA test to negative conversion (negative conversion of SARS-CoV-2 virus) in COVID-19 patients.

**Methods:**

This study included 116 hospitalized patients with COVID-19 from January 30, 2020 to March 4, 2020 in Wuhan, China. Throat swab samples were collected for qRT-PCR testing of SARS-CoV-2 RNA, and all patients included in this study were positive for this test.

**Results:**

The multivariate Cox proportional hazards model showed that disease severity (HR = 0.572; 95% CI 0.348–0.942; *p* = 0.028) was a protective factor for the time from symptom onset to positive nucleic acid detection. Meanwhile, the time from symptom onset to positive nucleic acid detection (HR = 1.010; 95% CI 1.005–1.020; *p* = 0.0282) was an independent risk factor for the delay in negative conversion time of SARS-CoV-2 virus. However, the severity of the disease (HR=1.120; 95% CI 0.771–1.640; *p* = 0.544) had no correlation with the negative conversion time of SARS-CoV-2 virus.

**Conclusions:**

Patients with more severe disease had a shorter time from symptom onset to a positive nucleic acid test. Prolonged time from symptom onset to positive nucleic acid test was an independent risk factor for the delay in negative conversion time of SARS-CoV-2 virus, and the severity of the disease had no correlation with negative conversion time of SARS-CoV-2 virus.

## Introduction

COVID-19 is a new infectious disease caused by a newly discovered coronavirus, severe acute respiratory syndrome coronavirus 2 (SARS-CoV-2). Since the outbreak in Wuhan in December 2019, the pandemic has spread to the world rapidly. At present, the number of new confirmed cases and deaths is increasing worldwide. As of February 23, a total of 424 million cases of laboratory-confirmed SARS-CoV-2 infection and more than 5.89 million deaths were registered worldwide (1). Detection of SARS-CoV-2 included etiological and serological examination of this virus. Among the etiological examination methods, the detection of SARS-CoV-2 nucleic acid by qRT–PCR is still the gold standard for the diagnosis of COVID-19 (2). Due to its capability of early diagnosis, high sensitivity and specificity, qRT–PCR is widely used in the fields of suspected case diagnosis, population screening, and health monitoring of staff (3). Two consecutive negative nucleic acid tests for respiratory pathogens (sampled ≥ 24 h apart) are one of the important criteria to define a patient's recovery. Therefore, negative conversion of SARS-CoV-2 RNA is essential to confirming whether a patient meets the criteria for discharge (4). Previous studies have shown that advanced age, more comorbid underlying diseases, fever and corticosteroid therapy use are risk factors for prolonged nucleic acid conversion (5, 6). However, only a few studies have explored the factors influencing the length of time from the onset of symptoms to the first nucleic acid test positive and the value of early positive nucleic acid test on the clinical outcome of patients. In this study, we retrospectively assessed the clinical characteristics of mild and severe COVID-19 patients in Wuhan, explored the factors associated with the length of time from symptom onset to the first positive nucleic acid test of throat swabs and investigated the effect of early positive nucleic acid test on the severity of disease and its significance in prognosis of patients, as well as the risk factors for prolonged conversion time from positive to negative nucleic acid test, to promote the early diagnosis and treatment of patients and improve the prognosis of patients.

## Materials and Methods

### Study Design and Subjects

We retrospectively analyzed the clinical characteristics of 116 laboratory-confirmed COVID-19 patients admitted to the East Campus of Renmin Hospital of Wuhan University from January 30, 2020 to March 4, 2020. The East Campus of Renmin Hospital of Wuhan University was one of the medical institutions designated for COVID-19 by the National Health Commission and responsible for the treatment of severe COVID-19 patients in China. In addition, retrospective analysis of clinical data posed no potential risk to the patients.

### Data Collection

We used a novel coronavirus 2019-nCoV nucleic acid detection kit (Easy Diagnosis Bio, Wuhan, China) to test respiratory specimens by qRT-PCR and all patients participating in this study showed positive test results. Patients under 18 years of age, pregnant women, and those transferred to other hospitals during hospitalization were excluded. Therefore, a total of 116 patients were included in this study. Patient demographics, laboratory test results, and the assessment of disease severity at admission were obtained from electronic medical records. Specifically, laboratory parameters included complete blood count (CBC), biochemical parameters, immune function, and coagulation function. According to the Protocol for Prevention and Control of COVID-19 (6th edition) issued by the National Health Commission, all patients met the clinical diagnostic criteria with the classification of disease severity (7). Two researchers independently reviewed the data collection forms to verify the accuracy of the data.

### Statistical Analysis

Continuous variables were expressed as the mean and standard deviation (SD) or median and interquartile range (IQR), and dichotomous variables were expressed as the number of cases and percentage (n,%). Normality tests were performed using the Shapiro-Wilk method. The *t*-test was used for the variables in normal distribution in the two groups of data, the wilcoxon rank sum test was used for non-normal distribution in the two groups of data; the analysis of variance was used for the variables that conform to the normal distribution in the four groups of data, the Kruskal–Wallis H test was used for the variables that do not conform to the normal distribution in the four groups of data, and Bonferroni correction method was used for pairwise comparison of the variables with different test results; Chi-square test was used for the dichotomous variables, and pairwise comparison was conducted for the variables with different test results. Univariate and multivariate analysis were used on the factors affecting the time from symptom onset to positive nucleic acid test and the conversion time from positive to negative nucleic acid test. Data were analyzed using R4.2 software, and *p* <0.05 was considered statistically significant.

## Results

### Association Between Clinical Characteristics and Laboratory Findings and the Time From Symptom Onset to the First Positive Nucleic Acid Test

As shown in Table 1, patients were divided into two groups according to the time from symptom onset to the first positive nucleic acid test. Seventy one patients who were diagnosed with positive nucleic acids within 1 week after onset were defined as the non-prolongation group. Of these 71 patients, 22 (30.99%) had severe disease and 49 (69.01%) had mild disease. The remaining 45 patients were diagnosed after more than 7 days and were defined as the prolongation group, in which the proportion of severe patients was 51.11% (23 patients), and the rate of severe disease in the prolongation group was significantly higher than that in the patients from the non-prolongation group (*p* <0.05). The demographics and laboratory findings were compared between the two groups. The mean age of the patients in the prolongation group was 61.53 years, which was higher than that in the non-prolongation group (56.46 years), but the difference was not statistically significant (*p* = 0.063). The median conversion time from a positive to a negative nucleic acid test in the prolongation group was 29 days (IQR, 16.0–43.5), which was significantly higher than that in the non-prolongation group (23 days, IQR, 15.0–37.75, *p* <0.05). In terms of routine blood examination, the lymphocyte count of patients in the prolongation group was significantly lower than that in the non-prolongation group; however, the comparison of other parameters between these groups showed no statistically significant difference (*p* > 0.05). Similarly, no significant difference in lymphocyte subsets or biochemical parameters (*p* > 0.05) was observed between the two groups.

**Table 1:** Comparison of clinical characteristics and laboratory findings between the two groups.

	** <7 days**	**≥7 days**	***P*** **-value**
**Sex, n (%)**			
Male	42 (59.15)	19 (42.22)	0.075
Female	29 (40.85)	26 (57.78)	
**Disease Severity, n (%)**			
Mild	49 (69.01)	22 (48.89)	0.027
Severe	22 (30.99)	23 (51.11)	
Age (years)	56.46 ± 13.71	61.53 ± 15.42	0.063
Median conversion time from positive to negative nucleic acid test (days)	23.00 (15.00, 37.75)	29.00 (16.00, 43.50)	0.041
White blood cells ( × 10^9^/L)	5.65 (4.50, 7.81)	5.65 (4.36, 7.11)	0.477
Neutrophils ( × 10^9^/L)	3.83 (2.61, 6.43)	3.89 (2.84, 5.75)	0.810
Lymphocytes ( × 10^9^/L)	1.12 (0.83, 1.55)	0.94 (0.64, 1.22)	0.027
Platelets ( × 10^9^/L)	219.34 ± 87.23	243.4 ± 85.61	0.137
CD3 + T cell counts (cells/uL)	683.00 (405.00, 926.00)	517 (315, 821)	0.091
CD4 + T cell counts (cells/uL)	380.00 (237.00, 626.00)	346 (202, 491)	0.179
CD8 + T cell counts (cells/uL)	229.00 (125.00, 336.00)	169.00 (101.50, 270.50)	0.084
CD4 + /CD8 + ratio	1.85 (1.23, 3.05)	1.85 (1.37, 2.82)	0.636
CD19 + B cell counts (cells/uL)	159.00 (106.00, 250.00)	132.00 (95.00, 199.00)	0.201
D-dimer (μg/mL)	0.82 (0.40, 2.61)	0.95 (0.43, 3.68)	0.713
Albumin (g/L)	35.92 ± 4.28	35.48 ± 3.79	0.561
Alanine aminotransferase (U/L)	28.00 (16.00, 50.00)	27.00 (19.00, 42.00)	0.715
Aspartate aminotransferase (U/L)	30.00 (21.00, 48.00)	30.00 (19.00, 40.00)	0.281
Alkaline phosphatase (U/L)	60.00 (51.00, 79.00)	64.00 (50.00, 92.00)	0.521
γ-glutamyl transpeptidase (U/L)	34.00 (19.00, 69.00)	30.00 (21.00, 56.00)	0.678
Total bilirubin (μmol/L)	10.70 (8.90, 13.80)	10.70 (7.70, 15.75)	0.923
Serum creatinine (μmol/L)	61.00 (50.00, 72.00)	63.00 (50.00, 71.00)	0.894
Lactate dehydrogenase (U/L)	266.00 (203.00, 391.00)	298.00 (229.00, 370.00)	0.682
C-reactive protein (mg/L)	37.50 (17.20, 73.60)	37.50 (11.95, 59.65)	0.463

**Figure 1:**
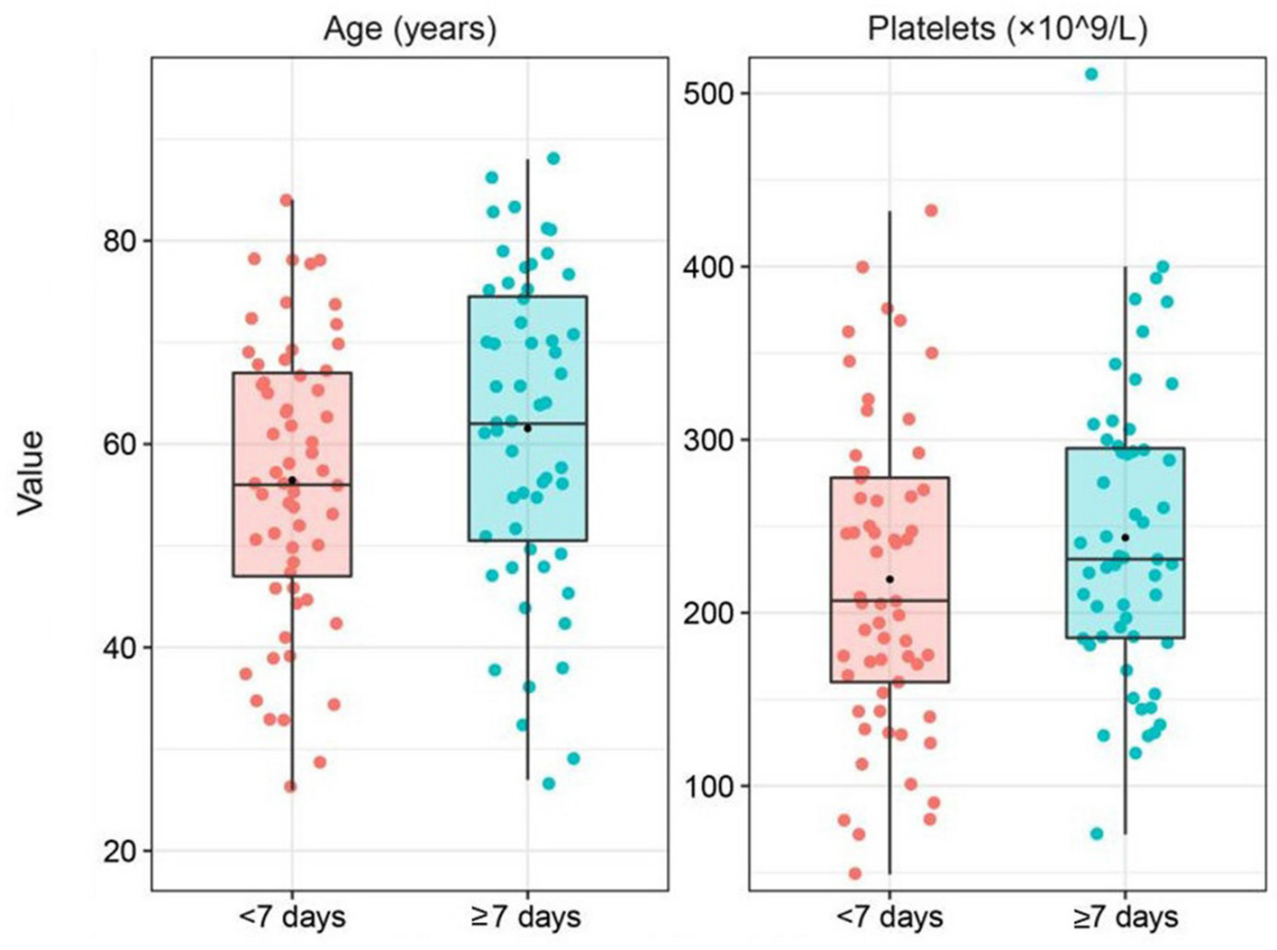
Box plot of variables grouped by the time from symptom onset to positive nucleic acid test was combined with the results of the univariate cox regression analysis and expertise.

As shown in Table 2 and Figure 1, univariate Cox regression analysis indicated that disease severity (*p* = 0.044), age (*p* = 0.04), and platelet (PLT) count (*p* = 0.035) were protective factors for the time from symptom onset to a positive nucleic acid test. To systematically analyze the factors affecting the length of time from symptom onset to first positive nucleic acid in patients, disease severity, age and platelet count were included in the multivariate Cox regression model as independent variables based on the analysis results of univariate Cox regression and our expertise on COVID-19. Multivariate analysis showed that disease severity (HR = 0.572; 95% CI 0.348–0.942; *p* = 0.028) was a protective factor for the time from symptom onset to a positive nucleic acid test.

**Table 2:** Univariate and multivariate cox regression model analysis results of the time from symptom onset to positive nucleic acid test.

	**Univariate model**		**Multivariate model**	
	**HR (95% CI)**	***P*** **-value**	**HR (95% CI)**	***P-*** **value**
Disease severity	0.675 (0.461–0.989)	0.044	0.572 (0.348–0.942)	0.028
Sex	0.756 (0.520–1.100)	0.142		
Age	0.988 (0.976–0.999)	0.040	1.010 (0.995–1.030)	0.192
White blood cells	0.990 (0.935–1.050)	0.732		
Neutrophils	1.020 (0.997–1.030)	0.104		
Lymphocytes	1.070 (0.743–1.530)	0.730		
Platelets	0.998 (0.995–1.000)	0.035	0.999 (0.996–1.000)	0.307
CD3 + T cell counts	1.000	0.801		
CD4 + T cell counts	1.000 (0.999–1.000)	0.912		
CD8 + T cell counts	1.000(0.999–1.000)	0.515		
CD4 + /CD8 + ratio	0.947 (0.801–1.120)	0.526		
CD19 + B cell counts	1.000 (0.999–1.000)	0.260		
D-dimer	1.000 (0.989–1.010)	0.924		
Alanine aminotransferase	1.000 (0.997–1.010)	0.416		
Aspartate aminotransferase	1.010 (0.998–1.020)	0.142		
Alkaline phosphatase	0.996 (0.990–1.000)	0.245		
γ-glutamyl transpeptidase	1.000 (0.998–1.000)	0.467		
Albumin	1.010 (0.965–1.060)	0.621		
Total bilirubin	0.985 (0.955–1.020)	0.329		
Serum creatinine	1.000 (0.994–1.01)	0.616		
Lactate dehydrogenase	1.000 (0.999–1.000)	0.905		
C-reactive protein	0.997 (0.994–1.000)	0.097		

### Association Between Clinical Characteristics and Laboratory Findings and the Length of Negative Conversion Time of SARS-CoV-2 Virus in Patients

As shown in Table 3, patients were divided into four groups according to the length of conversion time from the first positive to negative nucleic acid test. Of all the patients in these groups, 23 experienced <2 weeks of this conversion time, 40 experienced 2–4 weeks, 26 experienced 4–6 weeks, and the remaining 27 experienced more than 6 weeks. The basic characteristics of patients and laboratory findings were compared among these groups, which showed that the most common symptom on admission was fever (75.86%), followed by cough (62.93%), fatigue (21.55%), shortness of breath (21.55%), and diarrhea (10.34%) dyspnea (9.48%). The most common comorbidity was hypertension (25.86%), followed by diabetes (16.38%) and cardiovascular disease (12.07%); however, no significant differences were found in these symptoms and comorbidities among these groups (*p* > 0.05).

**Table 3:** Comparison of clinical characteristics and laboratory findings among the four groups.

	** <2 weeks**	**2 ~ 4 weeks**	**4 ~ 6 weeks**	**≥6 weeks**	***P*** **-value**
Sex, n (%)					0.880
Male	12 (52.17)	23 (57.50)	13 (50.00)	13 (48.15)	
Female	11 (47.83)	17 (42.50)	13 (50.00)	14 (51.85)	
Disease severity, n (%)					0.709
Mild	13 (56.52)	26 (65.00)	14 (53.85)	18 (66.67)	
Severe	10 (43.48)	14 (35.00)	12 (46.15)	9 (33.33)	
Age (years)	64.74 ± 14.32	56.15 ± 14.58	60.73 ± 14.8	56.07 ± 14.1	0.091
**Symptoms and signs, n (%)**					
Fever	17 (73.91)	29 (72.5)	19 (73.08)	23 (85.19)	0.640
Cough	15 (65.22)	28 (70.00)	12 (46.15)	18 (66.67)	0.240
Shortness of breath	8 (34.78)	6 (15.00)	8 (30.77)	3 (11.11)	0.092
Dyspnea	2 (8.70)	2 (5.00)	3 (11.54)	4 (14.81)	0.578
Fatigue	8 (34.78)	5 (12.50)	7 (26.92)	5 (18.52)	0.178
Diarrhea	3 (13.04)	4 (10.00)	3 (11.54)	2 (7.41)	0.924
**Comorbidities, n (%)**					
Hypertension	8 (34.78)	6 (15.00)	7 (26.92)	9 (33.33)	0.239
Diabetes	5 (21.74)	7 (17.50)	4 (15.38)	3 (11.11)	0.781
Cardiovascular and cerebrovascular disorders	4 (17.39)	2 (5.00)	4 (15.38)	4 (14.81)	0.398
Other comorbidities	10 (43.48)	19 (47.50)	10 (38.46)	7 (25.93)	0.346
Median time from symptom onset to positive nucleic acid test (days)	4 (0, 8)	5 (2.5, 8.25)	6.5 (3.5, 10)	9 (6, 14.5)^a^	0.007
Median time from onset of symptom to hospital admission (days)	7 (5.5, 13)	10 (7, 10.5)	12 (8.25, 14.75)	12 (8.5, 14.5)^b^	0.020
White blood cells ( × 10^9^/L)	5.51 (4.37, 7.70)	5.37 (4.42, 6.74)	6.23 (4.61, 10.06)	5.65 (4.49, 7.12)	0.507
Neutrophils ( × 10^9^/L)	3.89 (2.98, 5.86)	3.46 (2.74, 5.08)	4.38 (2.93, 9.16)	3.83 (2.44, 5.83)	0.481
Lymphocytes ( × 10^9^/L)	1.05 ± 0.44	1.14 ± 0.61	1.06 ± 0.46	1.16 ± 0.58	0.816
Platelets ( × 10^9^/L)	231.70 ± 74.90	229.38 ± 100.81	220.92 ± 76.62	241.44 ± 87.01	0.864
CD3 + T cell counts (cells/uL)	510.00 (323.50, 854.50)	602.50 (358.70, 927.20)	631.00 (342.50, 830.00)	670.00 (435.00, 926.50)	0.818
CD4 + T cell counts (cells/uL)	381.00 (217.50, 495.00)	352.50 (221.00, 551.00)	377.00 (220.00, 562.00)	443.00 (216.00, 528.00)	0.960
CD8 + T cell counts (cells/uL)	118.00 (91.50, 261.00)	249.00 (129.75, 337.50)	194.00 (76.00, 305.50)	207.00 (134.00, 319.00)	0.151
CD4 + /CD8 + ratio	2.53 (1.62, 3.40)	1.60 (1.07, 2.16)^a^	2.44 (1.26, 3.23)	1.85 (1.20, 3.09)	0.037
CD19 + B cell counts (cells/uL)	143.00 (113.00, 212.50)	118.00 (87.00, 177.25)	156.00 (86.00, 245.25)	159.00 (104.50, 273.50)	0.495
D-dimer (μg/mL)	1.05 (0.62, 4.51)	0.70 (0.36, 2.42)	0.84 (0.36, 3.21)	0.82 (0.48, 2.70)	0.370
Alanine aminotransferase (U/L)	27.00 (20.50, 42.50)	29.00 (16.75, 42.00)	24.50 (16.75, 50.75)	27.00 (18.00, 44.00)	0.966
Aspartate aminotransferase (U/L)	30.00 (20.00, 45.00)	29.50 (20.75, 39.25)	31.50 (19.25, 43.00)	34.00 (19.50, 45.00)	0.938
Alkaline phosphatase (U/L)	77.00 (57.00, 90.50)	59.00 (48.75, 84.50)	59.00 (50.50, 72.75)	65.00 (51.00, 86.50)	0.264
γ-glutamyl transpeptidase (U/L)	40.00 (20.00, 57.00)	30.50 (21.00, 68.25)	31.00 (20.25, 38.00)	31.00 (20.00, 57.00)	0.925
Total bilirubin (μmol/L)	11.30 (8.05, 16.05)	10.50 (7.83, 16.43)	10.90 (8.32, 13.57)	10.30 (8.80, 14.00)	0.960
Serum creatinine (μmol/L)	65.00 (55.50, 77.50)	63.50 (49.17, 70.00)	56.50 (47.25, 69.50)	59.00 (49.00, 69.00)	0.571
Lactate dehydrogenase (U/L)	300.00 (230.50, 381.50)	261.50 (206.70, 328.20)	307.50 (244.00, 378.00)	242.00 (210.50, 442.00)	0.454
C-reactive protein (mg/L)	50.90 (28.70, 92.45)	37.5 (17.18, 62.73)	37.50 (5.78, 63.09)	37.5 (17.00, 48.10)	0.213

a *Indicates p < 0.05 in the pairwise comparison with the <2 weeks group*.

b *Indicates p < 0.05 when compared with the 2 to 4 weeks group*.

The median time from symptom onset to a positive nucleic acid test was 9 days (IQR, 6.0–14.5) in patients with a conversion time of more than 6 weeks from a positive to a negative nucleic acid test, which was significantly longer than that in patients with a conversion time of <2 weeks (4 days, IQR, 0–8.0) (*p* < 0.05). In addition, patients with a conversion time of more than 6 weeks had a longer disease duration before admission than patients with a conversion time of 2 to 4 weeks (*p* < 0.05). In terms of routine blood examination, there were no statistically significant differences in white blood cell count, neutrophil count or lymphocyte count among these groups (*p* > 0.05). For immunological parameters, the CD4/CD8 ratio was lower in patients with conversion times of 2 to 4 weeks than in patients with conversion times of <2 weeks (*p* < 0.05). However, CD3+ T cell and CD4+ T cell counts did not show statistically significant differences between these groups (*p* > 0.05).

**Figure 2:**
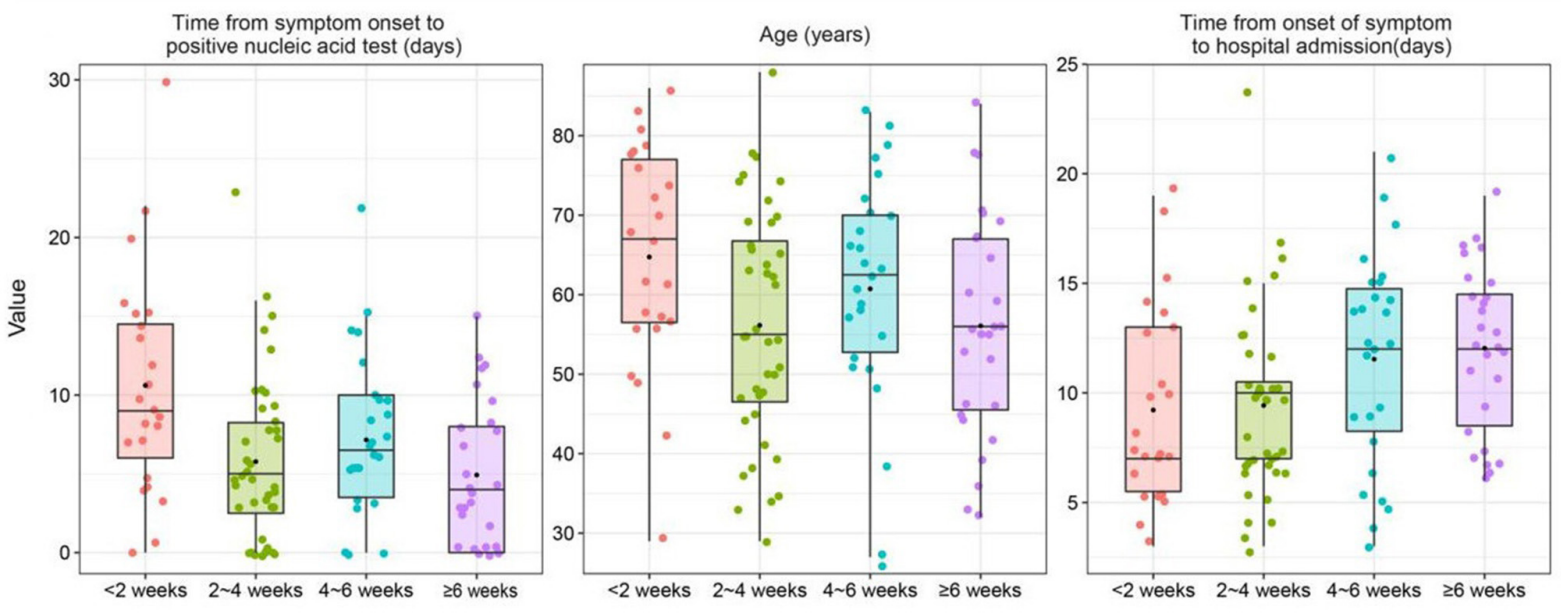
Box plot of variables grouped by the conversion time from positive to negative nucleic acid test was combined with the results of the univariate cox regression analysis and expertise.

As shown in Table 4 and Figure 2, univariate Cox regression analysis indicated that the time from symptom onset to a positive nucleic acid test (*p* = 0.019) and disease duration before admission (*p* = 0.004) were significantly correlated with the negative conversion time of SARS-CoV-2 virus. Combined with the results of univariate Cox regression analysis and our expertise in COVID-19, the time from symptom onset to positive nucleic acid test, duration of disease before admission and age were included as independent variables in the multivariate Cox regression model, and the results showed that the time from symptom onset to positive nucleic acid test (HR = 1.010; 95% CI 1.005–1.020; *p* = 0.0282) was an independent risk factor for prolonged negative conversion time from positive to negative nucleic acid test.

**Table 4:** Univariate and multivariate cox regression model analysis results of the conversion time from positive to negative nucleic acid test.

	**Univariate model**		**Multivariate model**	
	**HR (95% CI)**	***P-*** **value**	**HR (95% CI)**	***P*** **-value**
Disease severity	1.120 (0.771–1.640)	0.544		
Sex	0.769 (0.528–1.120)	0.172		
Age	1.010 (0.996–1.020)	0.179	1.000 (0.984–1.020)	0.192
Time from symptom onset to positive nucleic acid test	1.040 (1.010–1.080)	0.019	1.010 (1.005–1.020)	0.028
Time from onset of symptom to hospital admission	0.937 (0.896–0.980)	0.004	1.000 (0.944–1.060)	0.602
Fever	0.725 (0.471–1.120)	0.145		
Cough	0.857 (0.582–1.260)	0.436		
Shortness of breath	1.310 (0.836–2.040)	0.241		
Dyspnea	0.754 (0.403–1.41)	0.376		
Fatigue	1.090 (0.700–1.700)	0.698		
Diarrhea	1.080 (0.586–1.980)	0.813		
Hypertension	1.000 (0.659–1.530)	0.990		
Diabetes	1.510 (0.922–2.490)	0.101		
Other comorbidities	1.140 (0.774–1.670)	0.514		
White blood cells	1.01 (0.949–1.070)	0.808		
Neutrophils	1.000 (0.985–1.020)	0.865		
Lymphocytes	0.869 (0.615–1.230)	0.426		
Platelets	0.999 (0.997–1.000)	0.251		
CD3 + T cell counts	1.000 (0.999–1.000)	0.504		
CD4 + T cell counts	1.000 (0.999–1.000)	0.457		
CD8 + T cell counts	1.000 (0.999–1.000)	0.646		
CD4 + /CD8 + ratio	0.999 (0.865–1.150)	0.984		
CD19 + B cell counts	1.000 (0.999–1.000)	0.560		
D-dimer	1.000 (0.989–1.010)	0.869		
Alanine aminotransferase	1.000 (0.995–1.010)	0.944		
Aspartate aminotransferase	0.998 (0.989–1.010)	0.604		
Alkaline phosphatase	1.000 (0.994–1.010)	0.767		
γ-glutamyl transpeptidase	0.999 (0.995–1.000)	0.492		
Albumin	1.000 (0.959–1.050)	0.842		
Total bilirubin	1.010 (0.977–1.040)	0.664		
Serum creatinine	0.999 (0.995–1.000)	0.513		
Lactate dehydrogenase	1.000 (0.999–1.000)	0.803		
C-reactive protein	1.000 (1.000–1.010)	0.078		

### Association Between Clinical Characteristics and Laboratory Findings and Disease Severity in Patients

As shown in Table 5, the relevant parameters between mild and severe patients were compared. The mean age of severe patients was 70.49 years, which was significantly higher than that of mild patients (51.49 years, *p* < 0.05). Among the mild patients, 50.70% were male, slightly lower than that in severe patients (55.56%), but the difference between the two groups was not statistically significant. The median time from symptom onset to the first positive nucleic acid test was 5.0 days (IQR, 2.5–8.0) in mild patients and significantly shorter than 8 days (IQR, 4.0–12.0) in severe patients (*p* = 0.047). The median conversion time from the first positive to negative nucleic acid test was 24 days (IQR, 15.5–41.5) and 25 days (14.0–38.0) in mild and severe patients, respectively (*p* = 0.671). In terms of routine blood examination, the white blood cell count and neutrophil count were increased to varying degrees, and the lymphocyte count was decreased (*p* < 0.05) in severe patients compared with mild patients. For the immunological indicators, CD3+ T cells and CD8+ T cells were significantly lower in severe patients (*p* < 0.05) than in mild patients, and there were no statistically significant differences in CD4+ T cells between the two groups (*p* > 0.05). In terms of the biochemical parameters, albumin concentration was significantly higher in mild patients than in severe patients (*p* < 0.05). Alanine aminotransferase (ALT), aspartate aminotransferase (AST), alkaline phosphatase (ALP) and γ-glutamyl transpeptidase (γ-GGT) in severe patients were slightly higher than those in mild patients, but the difference was not statistically significant between the two groups (*p* > 0.05).

**Table 5:** Comparison of clinical characteristics and laboratory findings between mild and severe patients with COVID-19.

	**Mild**	**Severe**	***P-*** **value**
**Sex, n (%)**			
Male	36 (50.70)	25 (55.56)	0.610
Female	35 (49.30)	20 (44.44)	
Age (years)	51.49 ± 12.30	70.49 ± 9.90	<0.001
Length of hospital stay (days)	37.00 (23.50, 46.00)	40 (31, 47)	0.309
Median time from symptom onset to positive nucleic acid test (days)	5 (2.5, 8)	8 (4, 12)	0.047
Median conversion time from positive to negative nucleic acid test (days)	24 (15.5, 41.5)	25 (14, 38)	0.671
Median time from onset of symptom to hospital admission (days)	10 (7, 14)	10 (7, 14)	0.776
White blood cells ( × 10^9^/L)	5.48 (4.13, 6.82)	6.49 (4.75, 9.00)	0.026
Neutrophils ( × 10^9^/L)	3.45 (2.43, 4.70)	4.88 (3.26, 7.47)	0.002
Lymphocytes ( × 10^9^/L)	1.14 (0.80, 1.55)	0.93 (0.61, 1.13)	0.009
Platelets ( × 10^9^/L)	235.27 ± 89.86	223.62 ± 82.58	0.484
CD3 + T cell counts (cells/uL)	684.00 (431.50, 958.50)	503.00 (306.00, 723.00)	0.011
CD4 + T cell counts (cells/uL)	394.00 (223.50, 613.50)	333.00 (197.00, 476.00)	0.119
CD8 + T cell counts (cells/uL)	259.00 (130.50, 354.00)	132.00 (96.00, 222.00)	0.001
CD4 + /CD8 + ratio	1.66 (1.15, 2.66)	2.16 (1.47, 3.33)	0.020
CD19 + B cell counts (cells/uL)	158.00 (106.00, 248.50)	137.00 (85.00, 193.00)	0.150
D-dimer (μg/mL)	0.59 (0.34, 1.42)	1.63 (0.71, 5.36)	<0.001
Albumin (g/L)	37.14 ± 3.97	33.47 ± 3.04	<0.001
Alanine aminotransferase (U/L)	27.00 (1800, 45.50)	28.00 (18.00, 45.00)	0.823
Aspartate aminotransferase (U/L)	30.00 (20.00, 40.00)	31.00 (20.00, 44.00)	0.509
Alkaline phosphatase (U/L)	59.00 (49.00, 78.50)	67.00 (52.00, 92.00)	0.106
γ-glutamyl transpeptidase (U/L)	31.00 (19.50, 60.50)	32.00 (21.00, 68.00)	0.465
Total bilirubin (μmol/L)	9.90 (7.60, 12.55)	13.10 (9.50, 18.30)	0.003
Serum creatinine (μmol/L)	58.00 (46.50, 68.00)	67.00 (57.00, 75.00)	0.005
Lactate dehydrogenase (U/L)	251.00 (208.50, 325.00)	324.00 (242.00, 404.00)	0.019
C-reactive protein (mg/L)	37.50 (13.05, 56.70)	41.80 (17.20, 89.50)	0.061

## Discussion

In this study, we retrospectively analyzed the clinical characteristics of 116 laboratory-confirmed COVID-19 patients and explored the factors associated with the length of time from symptom onset to the first positive nucleic acid test of throat swabs, as well as the risk factors for prolonged conversion time from positive to negative nucleic acid test. We concluded that the prolonged time from symptom onset to positive nucleic acid detection was an independent risk factor for the delay in SARS-CoV-2 RNA negative conversion time.

The strategy of comprehensive and scientific detection of SARS-COV-2 in specific populations and environments through qRT–PCR provides support for COVID-19 pandemic prevention and control in China (8, 9). Nucleic acid screening helps identify infected individuals in a timely manner and prevents the spread of this pandemic (10). Nucleic acid detection of SARS-CoV-2 virus by qRT–PCR is considered the gold standard for COVID-19 diagnosis (2). At present, throat swab samples are mainly used for the diagnosis of suspected cases, population screening and staff health monitoring, but the results may be affected by the patient's viral load, the quality of samples, the mutation and recombination of SARS-CoV-2 and other factors, leading to false negative results (11, 12). Younger patients are more likely to have false negative results in the early stages of the disease (13). Recently, the emergence of new SARS-CoV-2 mutant strains have been associated with a surge in the number of infections worldwide (14). Mutations and recombination which lead to the emergence of novel lineages of SARS-CoV-2 can reduce the sensitivity of qRT–PCR and cause false negative results in throat swab detection (12). Meanwhile, the Protocol for Prevention and Control of COVID-19 (6th edition) states that negative nucleic acid test results do not rule out SARS-CoV-2 virus infection (7). Several studies have reported that qRT–PCR produces false negative results, including one study from Beijing that reported a case with consecutive false negative qRT–PCR results (15). Our study found that patients who tested positive for nucleic acids within a week from the onset of symptoms had a significantly lower rate of severe disease than those who tested more than a week later. Therefore, there is a need to develop highly sensitive and specific test methods to improve qRT–PCR assays and serological analysis to reduce false negative results and promote timely diagnosis and eventually to reduce the rate of severe patients (16). At the same time, our study found that disease severity was a protective factor for the time from symptom onset to a positive nucleic acid test: the more severe the patients' disease was, the shorter the time from symptom onset to a positive nucleic acid test. The reason may be related to the significantly higher viral load in severe patients than in mild patients (17). Studies have reported that the viral load of nasopharyngeal swabs in severe patients can even reach 60 times that of mild patients (18). However, a retrospective study from New York University found that the initial viral load was significantly higher in mild COVID-19 patients than in severe patients who required hospitalization (19). In the case of a PCR test, it is not only worth considering whether the patient's result is positive, but also the degree of viral load (20). Further studies are needed to further investigate the relationship between disease severity and viral load in COVID-19 patients.

The COVID-19 prevention and control protocol was based on the detection of SARS-CoV-2 nucleic acid in samples of respiratory tract or blood by real-time fluorescent qRT–PCR to determine diagnostic and discharge criteria for patients (21). However, there is no consistent standard to accurately define the duration of SARS-CoV-2 virus infection in the diagnosis and treatment of this disease in an actual clinical environment. Only a few studies have analyzed the factors influencing the conversion time from positive to negative tests using throat swab samples for nucleic acid detection. Our study found that a prolonged time from symptom onset to a positive nucleic acid test was an independent risk factor for a prolonged negative conversion time of SARS-CoV-2 RNA. If patients were diagnosed in time, the severity of the disease can be effectively predicted, and the progression of the disease from mild to severe conditions can be reduced, which is of great clinical significance for the prevention and control of COVID-19. A retrospective cohort study from Wuhan, China, found that the median duration of viral shedding in recovered patients was 20.0 days, and the longest duration of viral shedding was 37.0 days (22). However, some of the infected individuals showed persistent positive nucleic acid test results. A study from Shanghai, China, reported that four COVID-19 cases with persistent positive nucleic acid tests had an average conversion time of 61 days from positive to negative nucleic acid tests (23). In our study, the median conversion time from the first positive to negative nucleic acid test was 24 days and 25 days in mild and severe patients, respectively, and the conversion time in one of the severe patients was up to 75 days. Zhou et al. found that the duration of viral RNA shedding in the upper respiratory tract specimens from a 75-year-old male patient with COVID-19 reached 111 days (24). The long-term positive viral RNA test result may be due to the damage to the immune system, immune tolerance and escape, or mutation of the virus (23, 25). Further studies are still needed to verify whether COVID-19 will form a chronic carrier state in humans.

The condition of severe patients with COVID-19 is critical and can change rapidly. Our results demonstrated that the white blood cell count, neutrophil count, and lymphocyte count were significantly lower in the severe group than in the mild group, suggesting that early diagnosis and intervention are very important to reduce the risk of death caused by the tendency to severe condition transformation (26). A study comparing the virus shedding time between patients hospitalized in the ICU and those not hospitalized in the ICU showed that the duration of virus shedding time of blood, saliva, and nasal samples was longer in ICU patients than in non-ICU patients (27). The reason may be related to the fact that severe patients might be more likely to receive invasive mechanical ventilation (17, 28). However, Zheng et al. (29) and Zhou et al. (24) found no association between the virus shedding time and the severity of COVID-19. In this study, we divided the virus shedding time into two time periods: from symptom onset to a positive nucleic acid test and from a positive nucleic acid test to a negative nucleic acid test. Further studies revealed that there were no significant differences in the average length of hospital stay between these two groups. The severity of the disease did not affect the conversion time of the nucleic acid test or the length of hospital stay, which may be because there was no significant positive correlation between the copy number of SARS-CoV-2 viral RNA and the severity of the disease (30). Viral clearance of SARS-CoV-2 may depend mainly on the host's own immune status. Previous studies have shown that advanced age and corticosteroid therapy use are risk factors for prolonged nucleic acid conversion. Elderly COVID-19 patients often show impaired immunity, which reduces the body's ability to clear the virus (31). Glucocorticoid therapy may be effective in suppressing T lymphocyte-mediated immune responses (6, 32). In clinical practice, some severe COVID-19 patients have a short conversion time from positive to negative nucleic acid tests; however, some mild patients and asymptomatic patients with SARS-CoV-2 infection may have a longer conversion time from positive to negative nucleic acid tests due to insufficient immune responses (33). Further clinical and scientific studies are still needed to explore the effect of disease severity on the time of nucleic acid conversion of SARS-CoV-2 virus.

This study has several limitations. First, due to the limitations of early disease identification and the urgency of controlling COVID-19, patients who were admitted early often lack laboratory data, such as IL-6, IL-10, IFN-γ and viral load. Second, the sample size of this study was relatively small. In addition, the deceased patients usually present critically on admission, but our study focused on the factors affecting patients' nucleic acid conversion. Deceased patients were excluded from this study because they lacked time for nucleic acid conversion, which may introduce potential bias risks and limit the generality of the results. Moreover, due to the limitations of retrospective studies, the results may be affected by confounding factors that we cannot control or observe in our analysis. In future studies, more rigorous randomized prospective trials are needed to verify our results.

## Conclusions

Patients with more severe disease had a shorter time from symptom onset to a positive nucleic acid test. Prolonged time from symptom onset to positive nucleic acid test was an independent risk factor for the prolonged negative conversion time of SARS-CoV-2 virus, and the severity of the disease had no correlation with negative conversion time of SARS-CoV-2 virus. Therefore, we emphasize the importance of early diagnosis and intervention to reduce the risk of death caused by the transformation to severe condition. Recently, with the emergence of SARS-CoV-2 Omicron variant (B.1.1.529), patients may present with relatively mild or even unusual clinical symptoms. However, no studies have been reported on the factors affecting the conversion of nucleic acids in patients infected with the Omicron variant. The analysis of factors influencing the time to nucleic acid conversion of different SARS-CoV-2 variants still needs to be further confirmed by large-scale clinical studies.

## Data Availability Statement

The original contributions presented in the study are included in the article/Supplementary Material, further inquiries can be directed to the corresponding authors.

## Ethics Statement

The studies involving human participants were reviewed and approved by Medical Ethics Committee of Renmin Hospital of Wuhan University. Written informed consent for participation was not required for this study in accordance with the national legislation and the institutional requirements.

## Author Contributions

YZ and KY contributed to the conception and design of this research and approved the version to be submitted. XZ, LX, JZ, and FZ performed data analysis and interpretation and wrote the manuscript. LX, SW, and WZ contributed to data acquisition and inspection. All authors contributed to the article and approved the submitted version.

## Conflict of Interest

The authors declare that the research was conducted in the absence of any commercial or financial relationships that could be construed as a potential conflict of interest. The reviewer WX declared a shared affiliation with the authors XZ, JZ, and YZ to the handling editor at the time of review.

## Publisher's Note

All claims expressed in this article are solely those of the authors and do not necessarily represent those of their affiliated organizations, or those of the publisher, the editors and the reviewers. Any product that may be evaluated in this article, or claim that may be made by its manufacturer, is not guaranteed or endorsed by the publisher.

## Abbreviations

COVID-19: Coronavirus disease 2019
SARS-CoV-2: Severe acute respiratory syndrome coronavirus 2
WHO: World Health Organization
IQR: Interquartile range
qRT-PCR: quantitative real-time polymerase chain reaction.
