# Supplementary material for: The Value of Early Positive Nucleic Acid Test and Negative Conversion Time of SARS-CoV-2 RNA in the Clinical Outcome of COVID-19 Patients
Source: Front Med (Lausanne). 2022 Apr 28;9:826900. doi: 10.3389/fmed.2022.826900 (PMC9095906; doi:10.3389/fmed.2022.826900)
Supplement: Supplementary file 1 [file Data_Sheet_1.DOCX]

Statistical Analysis

Continuous variables were expressed as the mean and standard deviation (SD) or median and interquartile range (IQR), and dichotomous variables were expressed as the number of cases and percentage (n,%). Normality tests were performed using the Shapiro-Wilk method.The t test was used for the variables in normal distribution in the two groups of data, the wilcoxon rank sum test was used for nonnormal distribution in the two groups of data; the analysis of variance was used for the variables that conform to the normal distribution in the four groups of data, the Kruskal–Wallis H test was used for the variables that do not conform to the normal distribution in the four groups of data, and Bonferroni correction method was used for pairwise comparison of the variables with different test results; Chi-square test was used for the dichotomous variables, and pairwise comparison was conducted for the variables with different test results. Univariate and multivariate analysis were used on the factors affecting the time from symptom onset to positive nucleic acid test and the conversion time from positive to negative nucleic acid test. Data were analyzed using R4.2 software, and p< 0.05 was considered statistically significant.

①The normality test method was the Shapiro-Wilk test. The R code for the normality test is as follows：

data<-read.csv("data.csv")

#Check the normality

library(psych)

var<-c(2:27) #Specify which variables to test

zt<-data.frame()

for (j in 1:2){ #Normality test for different groups

data.1<-data[data$group==j,]

for(i in var){

fit <-shapiro.test(data.1[,i])

new<-cbind(j,names(data.1)[i],fit$statistic,fit$p.value)

zt<-rbind(zt,new)

}

}

names(zt)<-c("group","variable","W","P")

write.csv(zt,"normality test.csv") #Save normality test results

②The R code for Cox regression is as follows：

# Univariate cox regression

library(survival)

covariates <- c("degree","time_stage2","sex","age"," time from onset of symptom to hospital admission","hospitalstay","WBC","NEU","LYM","PLT","CD3","CD4","CD8","CD4.CD8","CD19","PT","D.dimer","ALT","AST","ALP","GGT","Alb","TBil","Scr","LDH","CRP1","PCT1")

univ_formulas <- sapply(covariates,

function(x) as.formula(paste('Surv(time_stage1, status)~', x)))

univ_models <- lapply( univ_formulas, function(x){coxph(x, data = data)})

univ_results <- lapply(univ_models,

function(x){

x <- summary(x)

p.value<-signif(x$wald["pvalue"], digits=3)

HR <-signif(x$coef[2], digits=3);

HR.confint.lower <- signif(x$conf.int[,"lower .95"], 3)

HR.confint.upper <- signif(x$conf.int[,"upper .95"],3)

HR <- paste0(HR, " (",

HR.confint.lower, "-", HR.confint.upper, ")")

coef <- signif(x$coef[1],digits = 3)

se <- signif(x$coef[3],digits = 3)

z <- signif(x$coef[4],digits = 3)

res<-c(coef,se,z,p.value,HR)

names(res)<-c("coef","se","z","p.value","HR (95% CI for HR)")

return(res)

})

res <- t(as.data.frame(univ_results, check.names = FALSE))

single_cox1 <- as.data.frame(res)

write.csv(single_cox1,"single_cox1.csv")

#Multivariate cox regression

res.cox <- coxph(Surv(time_stage1, status) ~ degree + time_stage2 + age+ time from onset of

symptom to hospital admission + PLT, data = data)

x <- summary(res.cox)

p.value=signif(as.matrix(x$coefficients)[,5],3)

HR=signif(as.matrix(x$coefficients)[,2],3)

low=signif(x$conf.int[,3],3)

high=signif(x$conf.int[,4],3)

coef=signif(as.matrix(x$coefficients)[,1],3)

se=signif(as.matrix(x$coefficients)[,3],3)

z=signif(as.matrix(x$coefficients)[,4],3)

res<-c(coef,se,z,p.value,HR)

names(res)<-c("coef","se","z","p.value","HR (95% CI for HR)")

multi_res=data.frame(coef=coef,se=se,z=z,

p.value=pvalue,

HR=paste(HR," (",low,"-",high,")",sep=""),

stringsAsFactors = F

)

write.csv(multi_res,"multi_cox1.csv")
